# Maternal Lutein Intake during Pregnancies with or without Gestational Diabetes Mellitus and Cognitive Development of Children at 2 Years of Age: A Prospective Observational Study

**DOI:** 10.3390/nu16020328

**Published:** 2024-01-22

**Authors:** Isma’il Kadam, Chauntelle Nebie, Mudar Dalloul, Joan Hittelman, Lawrence Fordjour, Lori Hoepner, Itamar D. Futterman, Howard Minkoff, Xinyin Jiang

**Affiliations:** 1 PhD Program in Biochemistry, Graduate Center of the City University of New York, New York, NY 10016, USA; ikadam@gradcenter.cuny.edu; 2 Department of Health and Nutrition Sciences, Brooklyn College of the City University of New York, Brooklyn, NY 11210, USA; chauntelle.r@gmail.com; 3 Department of Obstetrics and Gynecology, SUNY Downstate Health Sciences University, Brooklyn, NY 11203, USA; mudar.dalloul@downstate.edu (M.D.); howard.minkoff@downstate.edu (H.M.); 4 Department of Psychology, SUNY Downstate Health Sciences University, Brooklyn, NY 11203, USA; joan.hittelman@downstate.edu; 5 Department of Pediatrics, SUNY Downstate Health Sciences University, Brooklyn, NY 11203, USA; lawrence.fordjour@downstate.edu; 6 Department of Environmental and Occupational Health Sciences, SUNY Downstate Health Sciences University, Brooklyn, NY 11203, USA; lori.hoepner@downstate.edu; 7 Departments of Obstetrics and Gynecology, Division of Maternal Fetal Medicine, Maimonides Medical Center, Brooklyn, NY 11219, USA; idf8788@gmail.com

**Keywords:** lutein, carotenoids, gestational diabetes mellitus, cognitive development, fetal programming

## Abstract

Lutein and its isomer zeaxanthin serve as antioxidants and preserve cognitive function during aging. However, whether lutein/zeaxanthin (L + Z) exposure early in life improves cognitive development of children is rarely explored. It is also unknown whether gestational diabetes mellitus (GDM), characterized by heightened oxidative stress, affects lutein metabolism. This prospective longitudinal cohort study examined the differences in L + Z intake and metabolism, as well as the association between maternal L + Z intake and children’s cognitive development in GDM versus non-GDM pregnancies. Seventy-six pregnant women (*n* = 40 with GDM) were recruited between 25 and 33 weeks of gestation and dietary intakes were recorded. At delivery, cord blood was collected, and 2 years later, the Bayley III developmental test was conducted on a subset of children (*n* = 38). The results suggest that GDM reduced cord blood lutein levels at birth; L + Z intake during pregnancy was associated with better cognitive (β = 0.003, *p* = 0.001) and language (β = 0.002, *p* = 0.038) scoring of children at 2 years regardless of GDM status. In conclusion, maternal L + Z intake was positively associated with children’s developmental scores, regardless of GDM. More studies are needed to confirm such associations.

## 1. Introduction

Lutein and its isomer zeaxanthin are non-provitamin A carotenoids found in foods such as leafy greens, corn, and egg yolks [1]. Lutein is enriched in the human retina and brain, both having high rates of metabolism and abundant amounts of vulnerable lipids such as docosahexaenoic acid (DHA)-containing phospholipids on the cellular membrane that are susceptible to oxidative damage [2,3]. The antioxidant properties of lutein may serve as a mechanism that helps protect vulnerable nervous tissues [1]. Although lutein is not the most abundant carotenoid in the diet or circulation, its concentration proportion in the brain is much higher, and more so in the brains of infants, constituting 60% of the total accumulated carotenoids compared to 35% in the brains of older adults [4,5].

Lutein intake demonstrates moderate associations with serum lutein levels in children [6]. Lutein supplementation at higher doses consistently increases macular pigment ocular density (MPOD), an indicator of lutein accumulation in the retina across studies [6,7]. Serum lutein levels, MPOD, and brain lutein levels are all demonstrated to be associated with cognitive performance in older adults, although with varied degrees of certainty [4]. The results of two meta-analyses including 46 and 7 studies also suggest that with a dosage higher than 5 mg per day, lutein supplementation improves vision and cognition markers in adults [7,8].

However, whether a higher exposure to lutein early in life, such as during the prenatal period, may have lasting benefits on cognitive function is largely unexplored. During pregnancy, maternal circulating lutein levels were found to be correlated with cord blood concentrations [9]. Although it is not the most abundant dietary carotenoid, the transfer rate of lutein from mother to fetus is the highest among all carotenoids [10]. Lutein is the predominant carotenoid in the placenta and cord blood, possibly reflecting its significance for the developing nervous system. The Project Viva prospective cohort study suggested that among 1580 mother–child pairs, greater maternal lutein/zeaxanthin (L + Z) consumption during the first and second trimesters of pregnancy was associated with better verbal intelligence and behavior regulation ability in mid-childhood [11].

Pregnancies complicated with gestational diabetes mellitus (GDM), hyperglycemia occurring during pregnancy in previously euglycemic women, demonstrate increased oxidative stress and have been associated with lower cognitive test scores in early childhood [12,13,14,15]. It is largely unknown whether GDM affects lutein accumulation in maternal and fetal tissues and whether a higher L + Z exposure during the prenatal and early postnatal period may counter the negative impact of GDM on offspring cognitive function.

One objective of this prospective longitudinal study was to investigate how maternal L + Z intake during mid to late gestation was related to lutein concentrations in maternal and fetal compartments and whether GDM affected such associations at birth. Another objective was to determine whether prenatal and early postnatal exposure to L + Z was related to cognitive development at 2 years of age and whether a higher exposure would overcome the negative influence of GDM on cognitive function of children.

## 2. Materials and Methods

### 2.1. Study Population and Study Procedures

This study was conducted according to the guidelines of the Declaration of Helsinki. The study protocol was reviewed and approved by both the City University of New York (CUNY) Institutional Review Board (IRB) (approval ID: 2016-0331, 8 September 2023) and the SUNY Downstate Health Sciences University IRB (approval ID: 816786, 28 March 2023). Written informed consent was obtained from each participant before participation in the study.

In this prospective cohort study conducted from September 2016 to June 2019, pregnant women with (*n* = 40) or without GDM (*n* = 36) who attended the prenatal clinic at the State University of New York (SUNY) Downstate Health Sciences University in Brooklyn, NY, USA were recruited to study the association between prenatal nutrition exposure and the developmental outcomes of children. The detailed methodology has been published previously [16]. The inclusion criteria included English-speaking, over 21 years of age, gestational age between 25 and 33 weeks, and singleton pregnancy. Exclusion criteria included pre-pregnancy cardiometabolic diseases including diabetes, cardiovascular conditions, kidney disease, and liver disease. Conditions such as pre-eclampsia and infections that developed during pregnancy were not part of the exclusion criteria. However, we did not have participants who developed these conditions, although there were participants who developed an elevated blood pressure.

At enrollment, a trained research assistant conducted a structured face-to-face interview with the participant to collect demographic and medical information. Participants were instructed to fast overnight for eight hours, then 10 mL of blood was drawn to retrieve plasma, buffy coat, and serum [16]. In the following week, the research assistant called the participant by phone on two weekdays and one weekend day to obtain three 24-h dietary recalls. The multi-pass method by Harnack and colleagues for the dietary recall was used [17,18].

Immediately after delivery, cord venous blood and placenta samples were collected. Obstetric and birth outcome information including neonate sex, mode of delivery, and weight and length at birth was collected through medical chart review. A total of 21 cord blood and placenta samples were retrieved from the GDM group and 26 were retrieved from the non-GDM group [19]. Cord blood samples were centrifuged to retrieve plasma. Full-thickness placenta biopsies (0.5 × 0.5 cm) were collected from four virtually divided quadrants of each placental disk and fixed in RNAlater^®^ overnight (Thermo Scientific, Grand Island, NY, USA) [20]. All samples were stored at −80 °C until analysis.

The maternal–child dyads from the same cohort were invited to a follow-up visit at SUNY Downstate when the children reached 2 years of age. There were 20 non-GDM and 18 GDM dyads that completed the follow-up visit. Responses to a semi-quantitative food frequency questionnaire (FFQ) about the child’s food intake were collected via a structured interview with the mother [21]. A licensed psychologist then conducted the Bayley Scales of Infant and Toddler Development-III (Bayley III) test to assess the child’s development in the cognitive, language, motor, socio-emotional, and adaptive domains [22].

### 2.2. Dietary Analysis

To assess maternal nutrient intake, the 3-day dietary recall was analyzed with the Nutrition Data System for Research (NDSR) software (version 2019, University of Minnesota, Minneapolis, MN, USA). The average daily intakes of L + Z were calculated as the average daily consumption of L + Z over the three days of recall. Dietary recalls with extremely low (<500 kcal/day) or high (>5000 kcal/day) total energy intake were excluded in data analysis. For the FFQ of children, average daily intakes of L + Z were calculated as the average daily consumption from the sum of weekly consumption frequency of the nutrient-containing food × approximate portion of intake each time × L + Z concentration in the food. Overall diet quality was assessed using the Healthy Eating Index (HEI)-2015 [23,24,25].

### 2.3. Plasma Lutein Measurement

Maternal and venous cord blood plasma samples were sent out to Creative Proteomics (Shirley, NY, USA) for lutein level measurement with ultra-high performance liquid chromatography-mass spectrometry/mass spectrometry (UHPLC-MS/MS) using an ABSCIEX API 4000 tandem mass spectrometer (AB Sciex, Framingham, MA, USA) connected to a Waters Acquity UPLC (Waters, Milford, MA, USA). Each plasma sample (50 µL) was mixed with 150 µL of methanol, centrifuged, and filtered through a 0.22 μm membrane filter. The Waters Acquity UPLC BEH C18 column (2.1 × 100 mm 1.7 μm) coupled with a VanGuard precolumn (2.1 × 5 mm 1.7 μm) was used for liquid chromatography. The mobile phase A was pure water with 0.2% formic acid and the mobile phase B was acetonitrile with 0.1% formic acid. The column temperature was held at 40 °C and the injection volume was 5 μL. The gradient was from 98% B in 10 min, with a flow rate of 0.45 mL/min. The ESI positive mode was used for MS and the conditions were as follows: ion source temperature, 550 °C; CUR, 30 psi; IS, 5000V, and GS1 and GS2, 45 psi. The lutein peak was detected at *m*/*z* 568.6 and a substantial fragment was detected at *m*/*z* 476.3.

### 2.4. Plasma Lipid Measurement

Since the fat-soluble lutein was transported by lipoproteins, especially HDL, and GDM was demonstrated to alter the lipid profile and tended to decrease cord blood HDL in the current cohort [16], we also assessed blood lipid (TG, FFA, HDL-c, and LDL-c) levels. Maternal and cord plasma triglyceride (TG) levels were measured with the Cayman triglyceride colorimetric assay kit (Cayman, Ann Arbor, MI, USA); high-density lipoprotein cholesterol (HDL-c) and low-density lipoprotein cholesterol (LDL-c) were measured with the Abcam cholesterol assay kit (Abcam, Cambridge, MA, USA); and free fatty acids (FFA) were measured with the NEFA-HR(2) kit from Wako (Wako Diagnostics, Richmond, VA, USA).

### 2.5. Placental Gene Expression Measurement

Since the placenta is a critical organ that mediates maternal-to-fetal lipid and fat-soluble nutrient transport, we assessed the expression of a few lipid transport-related genes in the placenta: FATP1 is a major protein that mediates fatty acid transport through the placenta. CD36 mediates lutein uptake in tissue and fat transport in the placenta. LPL hydrolyzes TG to FFA to facilitate placental lipid uptake. Placental gene expression was measured with real-time quantitative PCR. RNA was extracted using the TRIzol^®^ reagent (Thermo Scientific). Reverse transcription was conducted using the High-Capacity cDNA Reverse Transcription kit (Thermo Scientific) following the manufacturer’s instructions. Gene transcript abundance was analyzed by means of quantitative real-time PCR with SYBR green detection using the CFX384 Touch™ Real-Time PCR Detection System (Bio-Rad, Hercules, CA, USA) as previously described [26]. Data were expressed as the fold difference of the gene of interest relative to the housekeeping gene glyceraldehyde 3-phosphate dehydrogenase (GAPDH) using the 2^−ΔΔCt^ method [27]. Primers for the genes of interest included cluster of differentiation 36 (CD36), forward 5′-CCTATTGGGAAAGTCACTGCGA-3′, reverse 5′-ACAGCATAGATTGACCTGCAAATA-3′; fatty acid transporter 1 (FATP1), forward 5′-GATGGCTATGTCAGCGAGAGCG-3′, reverse 5′-TGTAGCCCAGCTCATCCATCA-3′; lipoprotein lipase (LPL), forward 5′-GTCCGCGGGCTACACCAAAC-3′, reverse 5’-GCATGGGCTCCAAGGCTGTATC-3′. Primers for GAPDH were, forward 5′-TGTTGCCATCAATGACCCCTT-3′, reverse 5′-CTCCACGACGTACTCAGCG-3′. All primers were designed using GeneRunner Version 3.01 [28].

### 2.6. Bayley III Test

At the 2-year follow up, children’s neurodevelopmental status was assessed with the Bayley Scales of Infant and Toddler Development™ Screening Test Third Edition (Bayley-III™ Screening Test) by a trained child psychologist with both the child and caregiver present, in an ambulatory setting. This tool has been validated to have high internal consistency and inter-rater reliability [29]. Raw summary scores of the 3 domains, namely cognitive, language, and motor development, were computed according to the manual and transformed to composite scores.

### 2.7. Salivary Cortisol Measurement

Since cortisol is a stress hormone in the hypothalamic pituitary adrenal (HPA) axis and its chronic elevation was associated with reduced cognitive performance in previous studies [30], we collected saliva from children during the 2-year follow-up in the morning between 9 and 11 a.m. using the SalivaBio Children’s Swab (Salimetrics, State College, PA, USA). Salivary cortisol levels were measured with the Salimetrics salivary cortisol ELISA kit following manufacturer’s instructions.

### 2.8. Statistical Analysis

L + Z intake and lutein levels in the blood were compared between the GDM and non-GDM groups using the analysis of variance (ANOVA) test accounting for maternal age, parity, race/ethnicity, education levels, and child’s sex as initial covariates. The covariates were all removed after a stepwise process that excluded covariates not reaching a *p* < 0.1. To assess the association between maternal L + Z intake or circulating lutein level and children’s outcomes at birth and at the 2-year follow-up, the generalized linear model (GLM) was used. The nutrient intake/blood level was entered as the independent variable, and the outcome was entered as the dependent variable, adjusted for GDM status and potential confounders including maternal parity, race/ethnicity, education levels, blood pressure, child’s sex, and Healthy Eating Index (HEI)-2015 during pregnancy, as well as the two-way interaction between GDM and intake/circulating level. Additional confounders added to the model for children’s outcomes at 2 years were children’s L + Z intake, gestational age at birth, small-for-gestational-age birth, and maternal breastfeeding duration. To assess the association between children’s intake and outcomes, the GLM models included maternal L + Z intake as a covariate instead. If there was a trend of interaction between GDM status and nutrient intake/circulating level in their correlation with an outcome (*p* < 0.1), we conducted a secondary GLM analysis stratifying the data by GDM status. The covariates were also eliminated via a stepwise process if not having a *p* value less than 0.1. After the stepwise process, the final GLM models above had GDM and parity remaining in the model, besides the independent and dependent variable. Data that were not normally distributed were log-transformed before analyses. Data were presented as mean ± standard deviation (SD). A *p* value < 0.05 was considered significant. Data were analyzed with SPSS software (version 24, IBM Inc., Armonk, NY, USA).

## 3. Results

### 3.1. Maternal L + Z Intake and Circulating Levels Were Unaltered Yet Cord Blood Lutein Levels Were Reduced in GDM Pregnancies

The study flowchart was previously published [16] and an updated version with information about the 2-year follow-up is included in Supplemental Figure S1. The demographic and birth outcome information was also previously published [16]. We observed a 50% sample size attrition at the 2-year follow-up due in part to the COVID-19 pandemic, which resulted in high mobility of the cohort. We compared the differences between those who completed the 2-year follow-up versus those who did not, and did not find differences in GDM status, prepregnancy BMI, demographic characteristics and birth outcomes, except that those who completed the follow-up had a higher average maternal age (Table 1).

We compared maternal L + Z intake in GDM versus non-GDM pregnancies and did not find any significant difference. Maternal plasma lutein levels were also not altered by GDM status. However, venous cord plasma concentrations of lutein were significantly reduced in the GDM versus non-GDM pregnancies (*p* = 0.022) (Table 2).

### 3.2. Higher Maternal L + Z Intake Was Associated with Lower Blood Lutein Levels

Interestingly, maternal L + Z intake during pregnancy was negatively associated with maternal plasma lutein levels (β = −0.001, *p* = 0.017), regardless of GDM status. However, neither maternal L + Z intake (β = 0.001, *p* = 0.24) nor maternal plasma lutein levels (β = −0.026, *p* = 0.90) were associated with cord plasma lutein concentrations.

### 3.3. Plasma HDL-c and LDL-c Levels Were Not Associated with Lutein Levels

Since the fat-soluble lutein was transported by lipoproteins, especially HDL, we assessed whether the blood lipid (TG, FFA, HDL-c, and LDL-c) levels were associated with maternal and cord plasma lutein levels. However, we did not find any significant association between these blood lipids in maternal or cord plasma and lutein levels, except that maternal lutein levels tended to be negatively associated with cord blood TG (β = −0.057, *p* = 0.052) and FFA levels (β = −0.017, *p* = 0.052) (Supplemental Table S1).

### 3.4. Placental FATP1 Expression Was Associated with Cord Blood Lutein Levels Only in Non-GDM Pregnancies

We also assessed the association of cord plasma lutein levels with the expression of a few lipid transport-related genes in the placenta which mediate maternal-to-fetal lipid and fat-soluble nutrient transport. Interestingly, we found a trend of interaction between GDM status and the fatty acid transporter FATP1 expression in relation to cord lutein levels (*p* = 0.10). After stratifying the analysis by GDM status, we found a positive association between placental FATP1 expression and cord lutein levels in non-GDM pregnancies (β = 44.2, *p* = 0.016). However, this association was eliminated in GDM pregnancies (β = −9.2, *p* = 0.52). CD36, which was demonstrated to mediate lutein uptake in tissue and fat transport in the placenta (β = −1.32, *p* = 0.19), as well as LPL, which hydrolyzes TG to FFA to facilitate placental lipid uptake (β = 0.46, *p* = 0.74), were not associated with cord lutein levels.

### 3.5. There Were No Associations between Maternal L + Z Intake or Status and Neonatal Anthropometrics

We previously found that GDM increased the incidence of large-for-gestational-age (LGA) births in this cohort [16]. There was evidence in animals that lutein supplementation alleviated obesity in rodents fed a high-fat diet [31]. We thus examined the association between maternal L + Z intake or status and neonatal length and weight, but did not identify any significant associations (Supplementary Table S2).

### 3.6. L + Z Intake and Status during Pregnancy and Bayley Test Scores

We then conducted a 2-year follow-up on the mother and child dyads and found that maternal L + Z intake during pregnancy was positively associated with children’s Bayley test score in the language (β = 0.002, *p* = 0.038) and cognitive domains (β = 0.003, *p* = 0.001), but not in the motor domain (β = 0.001, *p* = 0.71) (Table 3). To determine whether the above correlations also applied to other carotenoids, we assessed the association between maternal β-carotene, lycopene, α-carotene, and total vitamin A intake and Bayley test scores, but did not find any significant associations. There were also no associations of maternal or cord blood lutein levels with children’s Bayley test scores (Table 3).

There were also no significant associations between children’s L + Z intake at 2 years of age and Bayley scores (Table 3).

### 3.7. Maternal L + Z Intake or Status Was Not Associated with Children’s Salivary Cortisol Levels

We also assessed the association between maternal L + Z intake or status during pregnancy and salivary stress hormone cortisol levels of children at the 2-year follow-up. Children’s salivary cortisol levels were 0.46 ± 0.60 µg/dL in the GDM and 0.38 ± 0.09 µg/dL in the non-GDM group. However, we did not find any associations between maternal L + Z intake or lutein levels and children’s cortisol levels (β = 0.001, *p* = 0.062 for maternal L + Z intake and β = −0.011, *p* = 0.17 for maternal plasma lutein levels).

## 4. Discussion

L + Z intake has been associated with positive cognitive function in adults [8,32,33], yet evidence regarding how early exposure to these carotenoids may be translated into positive outcomes in cognitive development is limited. In this study, we found that L + Z intake of mothers during pregnancy was associated with better development in both language and cognitive domains of children in early childhood. GDM may negatively influence lutein supply to the fetus, which could potentially have adverse effects on cognitive development.

Despite meta-analyses having demonstrated the overall positive effects of L + Z intake or supplementation on cognitive function in older adults [8], whether it is also critical for cognitive functions early in life is largely unknown. Lutein is preferably accumulated in various brain regions such as the hippocampus and the prefrontal, frontal, auditory, and occipital cortices, suggesting a neurotrophic role [5]. Lutein levels in human milk were associated with better recognition memory of 6-month-old infants in an observational study [34]. MPOD levels have been associated with the academic performance of young children [35]. The Project Viva cohort study demonstrated that maternal gestational intake of L + Z was related to better cognitive and behavioral scores in mid-childhood [11]. Our study’s results corroborate their findings of a positive relationship between prenatal L + Z exposure and the cognitive development of children, but at an earlier age. In addition, we found that early language development may also be improved with a higher L + Z exposure during the prenatal period. Moreover, other carotenoids such as β-carotene and lycopene were not associated with children’s Bayley scores. Although it is possible that mothers who had a higher L + Z intake during pregnancy might have a higher quality diet in general, while a previous study demonstrated that maternal dietary quality during pregnancy is positively associated with visual spatial skills at early childhood and intelligence and executive function at mid-childhood [36], the maternal HEI score which we used as a proxy of dietary quality was not associated with any Bayley measurements of children in the current study. Overall, the association with children’s neurodevelopmental measurements seems to be specific to maternal L + Z exposure. It is likely that the higher maternal L + Z intake promotes the transport of L + Z through the placenta to the fetus, where it may play several critical roles. First, it may serve as an antioxidant to reduce oxidative stress, which is important for the rapidly developing brain of the fetus, which has high rates of metabolism [37]. Preclinical studies have demonstrated that lutein protects neural tissues from oxidative stress injuries resulting from hydrogen peroxide and ischemia-reperfusion [38,39]. Second, it may protect DHA on the cellular membrane of neurons from peroxidation [40]. Third, it may affect gap junctional communication and permit the metabolic coupling of cells to jointly maintain homeostasis under stress [41,42].

GDM is a metabolically disturbed state during pregnancy characterized by higher oxidative stress and a reduction in DHA transfer to the fetus [43,44,45,46]. In this sense, increasing L + Z exposure may provide an ancillary solution to mitigate some of the negative influence of GDM on fetal development. We report for the first time that GDM is associated with a lower lutein level in the cord blood. This could potentially impair lutein accumulation in the fetal brain tissue, since lutein levels in the blood are associated with brain lutein levels according to a study in older adults [4]. We also found that GDM led to lower language scores at the 2-year follow-up of this cohort (unpublished data). A previous case–control study also demonstrated that children exposed to GDM prenatally had more language skill deficits [47]. GDM was also found to be associated with lower scores of psychomotor development in infants and lower intelligence quotient scores and educational attainment in school-age children [14,48]. As such, increasing L + Z intake of GDM pregnant mothers may be a solution to eliminate the negative influence of GDM on language and cognitive development of children.

We also tentatively explored whether the alteration in lipid profile mediated the interaction between GDM and lutein metabolism. GDM is often associated with dyslipidemia such as increases in plasma triglycerides, FFAs, and cholesterol [16,49,50]. Lutein is absorbed and transported by lipoproteins, mainly HDL, in the body, and the blood levels of lutein were reported to be positively associated with HDL levels [51]. However, we did not find any association between blood lutein and lipoproteins or other blood lipid markers in the current study, regardless of GDM status. This lack of association was unexpected but may be partly attributed to the relatively small sample size of this study. There is scarce evidence regarding how lutein is transported through the placenta, although using β-carotene as an example cross-placental transport of carotenoids may be facilitated by a series of lipid metabolic genes [52]. We did find that the mRNA expression of *FATP1*, which is a major fatty acid transporter in the placenta, was positively associated with cord blood lutein levels, but only in non-GDM pregnancies. While the positive association between placental *FATP1* expression and cord lutein levels is consistent with the concurrent increase in fat and fat-soluble nutrients, the dissociation of the two may have reflected the dysregulation of fat and fat-soluble nutrient transport during GDM, which may be a potential contributor to the lower fetal cord blood lutein levels in GDM pregnancies.

While lutein levels may be impacted by lipid metabolism, recent research suggests that lutein intake may also affect lipid metabolism by serving as an antioxidant to prevent peroxidation of lipoproteins and increasing peroxisome proliferators activated receptors (PPAR)-α gene expression, which promotes fatty acid catabolism, thereby reducing adiposity and improving cardiometabolic health [53,54]. Lutein supplementation was found to reduce the risk of obesity and glucose intolerance in high-fat-diet-fed rodents [31]. A major adverse outcome of GDM is fetal overgrowth or macrosomia at birth due partly to the excess fat accretion of the fetus [55]. However, our current study did not identify any association between maternal L + Z intake and offspring anthropometrics at birth. Nevertheless, higher maternal L + Z intake tended to be associated with lower TG and FFA in the cord blood, although not reaching statistical significance. Whether this result indicates a positive impact of maternal L + Z intake on fetal lipid metabolism requires further research in RCTs and observational studies with a larger sample size.

This study has some limitations, such as the lack of measurement of MPOD in the maternal and child dyad, which is a more reliable marker of tissue lutein status that correlates with intake [7]. Surprisingly, we found a negative association between maternal L + Z intake and circulating levels, possibly due to the relatively low L + Z intake of the cohort, since a positive association between L + Z intake and circulating level is more pronounced with higher doses of L + Z, adding to the heterogeneity of studies reporting on the relationship between L + Z intake and circulating levels [6,56,57,58,59]. We only performed blood lutein measurements, while concentrations of other carotenoids such as zeaxanthin may provide additional insights. The study also has a small sample size and a high dropout rate for the 2-year follow-up, although we included data at several levels to provide a comprehensive view of intake, status, and cognitive outcomes. There was no significant demographic and birth outcome characteristic difference between those who participated in the follow-up versus those who did not, except for maternal age. A larger sample size may have increased the statistical power to clarify the borderline differences between children’s L + Z intake and motor development scoring or maternal L + Z intake and children’s cortisol levels. Although this study provided a general assessment of development using Bayley III in the early childhood, more sophisticated assessments targeting a specific aspect of development and at later time points of childhood may provide further insights into the relationship between early lutein exposure and children’s developmental outcomes.

## 5. Conclusions

In conclusion, in this study we demonstrate that the L + Z intake of pregnant women has a positive association with cognitive and language development of children in early childhood. GDM decreased lutein levels in the cord blood, which may indicate the need for higher L + Z intake for women with GDM.

## Figures and Tables

**Table 1:** Maternal characteristics and birth outcomes of participants who completed the 2-year follow-up versus those who did not.

	Completed Follow-Up (*n* = 38)	Lost to Follow-Up (*n* = 38)	*p*
Maternal age (year)	33.4 ± 4.9	31.3 ± 5.4	0.027
Maternal BMI (kg/m^2^)	30.4 ± 7.1	31.9 ± 8.5	0.39
GDM diagnosis	18 (47%)	24 (63%)	0.37
First pregnancy (n, %)	9 (24%)	8 (21%)	0.43
Race/ethnicity (n, %)			0.13
Non-Hispanic white	1 (3%)	0 (0%)	
Non-Hispanic black	34 (89%)	33 (87%)	
Hispanic white	3 (8%)	2 (5%)	
Asian	0 (0%)	3 (8%)	
Maternal education level (n, %)			0.11
≤High school	24 (63%)	28 (74%)	
≥Some college	14 (37%)	10 (26%)	
Unemployment (n, %)	12 (32%)	12 (32%)	1
Cesarean section (n, %)	22 (58%)	20 (53%)	0.36
Pre-term delivery (n, %)	6 (16%)	7 (18%)	0.52
Sex (female) (n, %)	22 (58%)	23 (61%)	0.36
Small-for-gestational-age (n, %)	5 (13%)	5 (13%)	1
Large-for-gestational-age (n, %)	9 (24%)	2 (5%)	0.078
Birth weight (g)	3111 ± 552	3329 ± 715	0.15
Breastfeeding rate	0.75		
Exclusive breastfeeding rate	0.61		
Average breastfeeding duration (month)	3.1 ± 3.8		

Analyzed with Student’s *t* test for continuous variables and the χ-square test for categorical variables. Values are mean ± standard deviation (SD).

**Table 2:** Lutein intake or circulating levels in the GDM versus the non-GDM group.

	Non-GDM	GDM	*p* Value
Maternal lutein/zeaxanthin intake (mg)	2.1 ± 3.0	1.8 ± 1.8	0.58
Maternal plasma lutein (ng/mL)	57.6 ± 11.6	60.5 ± 8.5	0.26
Cord plasma lutein (ng/mL)	55.6 ± 20.4	44.4 ± 5.1	0.022

*n* = 40 for the GDM group and *n* = 36 for the non-GDM group for maternal biomarkers; *n* = 21 for the GDM group and *n* = 26 for the non-GDM group for cord blood markers. Analyzed with ANOVA. Values are mean ± standard deviation (SD).

**Table 3:** Associations between lutein intake or plasma levels and children’s Bayley test scores at 2 years of age.

	Cognitive		Language		Motor	
	β	*p* Value	β	*p* Value	β	*p* Value
Maternal lutein/zeaxanthin intake	0.002	0.038	0.003	0.001	0.001	0.71
Maternal α-carotene intake	0.002	0.31	−0.001	0.79	0.001	0.61
Maternal β-carotene intake	0.001	0.28	0.001	0.95	0.001	0.77
Maternal lycopene intake	0.001	0.51	0.001	0.83	0.001	0.18
Total vitamin A intake	0.001	0.33	0.001	0.93	0.001	1.0
Maternal plasma lutein	−0.02	0.92	−0.31	0.25	0.11	0.64
Cord plasma lutein	−0.15	0.47	−0.03	0.87	−0.26	0.17
Children’s lutein/zeaxanthin intake	0.001	0.99	0.001	0.96	0.001	0.074

*n* = 20 for the non-GDM and *n* = 18 for the GDM group. Analyzed with generalized linear model adjusted for GDM status and maternal parity.

## Data Availability

Data are contained within the article.

## Supplementary Materials

The following supporting information can be downloaded at: https://www.mdpi.com/article/10.3390/nu16020328/s1, Figure S1: Flowchart of the study. Supplementary Table S1. Association between blood lipid and lutein levels in the maternal and fetal dyad. Supplementary Table S2. Association between lutein intake or status and neonatal anthropometrics.
