# Supplementary material for: Maternal Lutein Intake during Pregnancies with or without Gestational Diabetes Mellitus and Cognitive Development of Children at 2 Years of Age: A Prospective Observational Study
Source: Nutrients. 2024 Jan 22;16(2):328. doi: 10.3390/nu16020328 (PMC10819807; doi:10.3390/nu16020328)
Supplement: Supplementary file 1 [file nutrients-16-00328-s001.zip › Table S1.pdf]

Supplementary Table S1. Association between blood lipid and lutein levels in the maternal and fetal dyad.

	Maternal lutein		Cord lutein	
	β	p value	β	p value
Maternal				
HDL	0.11	0.28	-0.11	0.38
LDL	-0.01	0.84	-0.04	0.19
TG	0.01	0.87	0.01	0.46
FFA	-0.01	0.71	-0.01	0.97
Cord				
HDL	0.097	0.49	-0.19	0.09
LDL	-0.059	0.26	-0.09	0.12
TG	-0.057	0.052	-0.02	0.64
FFA	-0.017	0.052	-0.01	0.59

n = 40 for the GDM group and n = 36 for non-GDM group for maternal biomarkers; n = 21 for the GDM group and n = 26 for the non-GDM group for cord blood markers. Analyzed with generalized linear model adjusted for GDM status. FFA, free fatty acid; HDL, high-density lipoprotein; LDL, low-density lipoprotein; TG, triglyceride.
