# Supplementary material for: Maternal Lutein Intake during Pregnancies with or without Gestational Diabetes Mellitus and Cognitive Development of Children at 2 Years of Age: A Prospective Observational Study
Source: Nutrients. 2024 Jan 22;16(2):328. doi: 10.3390/nu16020328 (PMC10819807; doi:10.3390/nu16020328)
Supplement: Supplementary file 1 [file nutrients-16-00328-s001.zip › Table S2.pdf]

Supplementary Table S2. Association between lutein intake or status and neonatal anthropometrics.

	Length		Weight		Head circumference	
	β	p	β	p	β	p
Maternal lutein/zeaxanthin intake	0.001	0.52	-0.033	0.28	0.001	0.97
Maternal plama lutein	-0.021	0.54	2.88	0.67	-0.006	0.84
Cord plasma lutein	0.009	0.36	-0.27	0.95	-0.035	0.24

n = 40 for the GDM group and n = 36 for non-GDM group for maternal biomarkers; n = 21 for the GDM group and n = 26 for the non-GDM group for cord blood markers. Analyzed with generalized linear model adjusted for GDM status and sex of neonate.
