# Supplementary figures and images for: Maternal Lutein Intake during Pregnancies with or without Gestational Diabetes Mellitus and Cognitive Development of Children at 2 Years of Age: A Prospective Observational Study
Source: Nutrients. 2024 Jan 22;16(2):328. doi: 10.3390/nu16020328 (PMC10819807; doi:10.3390/nu16020328)

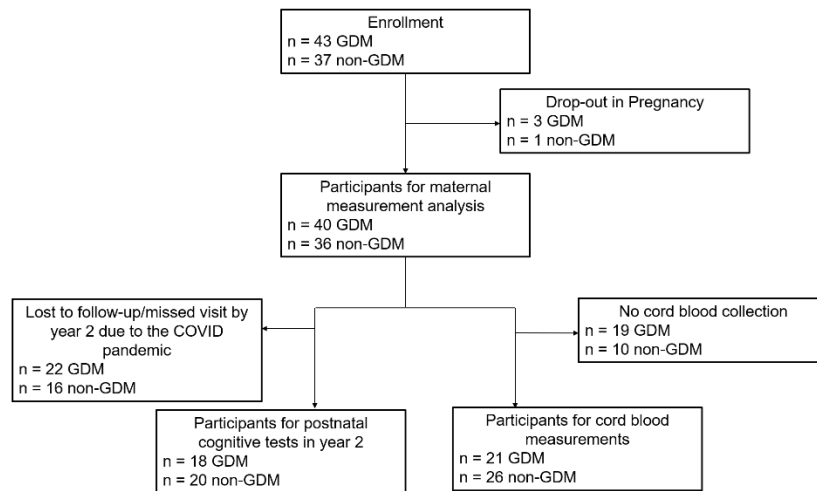

**Supplementary Figure S1.** Flowchart of the study.

Supplement: Supplementary file 1 [file nutrients-16-00328-s001.zip › Figure S1.pdf]
